# Prevalence of reflux nephropathy in Iranian children with solitary kidney: results of a multi-center study

**DOI:** 10.1186/s12882-022-02703-z

**Published:** 2022-02-21

**Authors:** Maryam Esteghamati, Hadi Sorkhi, Hamid Mohammadjafari, Ali Derakhshan, Simin Sadeghi-Bojd, Hossein Emad Momtaz, Masoumeh Mohkam, Baranak Safaeian, Nakysa Hooman, Afshin Safaeiasl, Mohsen Akhavan Sepahi, Khadijeh Ghasemi, Zahra Bazargani, Elham Emami

**Affiliations:** 1 Department of Pediatric Nephrology, Clinical Research Development Center of Children’s Hospital, Hormozgan University of Medical Sciences, Bandar Abbas, Iran; 2 Non-Communicable Pediatric Diseases Research Center, Health Research Institute, Babol University of Medical Sciences, Babol, Iran; 3 Pediatric Infectious Diseases Research Center, Communicable Diseases Institute, Mazandaran University of Medical Sciences, Sari, Iran; 4 Shiraz Nephro-Urology Research Center, Shiraz University of Medical Sciences, Shiraz, Iran; 5 Genetics of Non-Communicable Disease Research Center, Zahedan University of Medical Sciences, Zahedan, Iran; 6 Division of Pediatric Nephrology, Besat Hospital, School of Medicine, Hamadan University of Medical Sciences, Hamadan, Iran; 7 Pediatric Nephrology Research Center, Research Institute for Children’s Health, Shahid Beheshti University of Medical Sciences, Tehran, Iran; 8 Taleghani Pediatric Hospital, Golestan University of Medical Sciences, Gorgan, Iran; 9 Aliasghar Clinical Research Development Center, Department of Pediatrics, School of Medicine, Iran University of Medical Sciences, Tehran, Iran; 10 Department of Pediatrics, School of Medicine, Guilan University of Medical Sciences, Rasht, Iran; 11 Department of Pediatrics, School of Medicine, Qom University of Medical Sciences, Qom, Iran; 12 Department of Pediatric Nephrology, Bushehr University of Medical Sciences, Bushehr, Iran; 13 Clinical Research Development Unit, Department of Pediatrics, Valiasr Hospital,School of Medicine, Fasa University of Medical Sciences, Fasa, Iran; 14 Department of Pediatrics, Hajar Shaherkord University of Medical Sciences, Shahrekord, Iran

**Keywords:** Vesicoureteral reflux, Nephropathy, Children, Solitary kidney, Renal agenesis

## Abstract

**Background:**

Given the importance of the function of the remnant kidney in children with unilateral renal agenesis and the significance of timely diagnosis and treatment of reflux nephropathy to prevent further damage to the remaining kidney, we aimed to determine the prevalence of reflux nephropathy in this subgroup of pediatric patients.

**Methods:**

In general, 274 children referred to pediatric nephrologists in different parts of Iran were evaluated, of whom 199 had solitary kidney and were included in this cross-sectional study. The reasons for referral included urinary tract infection (UTI), abnormal renal ultrasonography, being symptomatic, and incidental screening. Demographic characteristics, including age and gender were recorded. History of UTI and presence of vesicoureteral reflux (VUR) were evaluated.

**Results:**

Of the 274 children evaluated in this study with the mean age (SD) of 4.71 (4.24) years, 199 (72.6%) had solitary kidney. Among these, 118 (59.3%) were male and 81 (60.7%) were female, 21.1% had a history of UTI, and VUR was present in 23.1%. The most common cause of referral was abnormal renal ultrasonography (40.2%), followed by incidental screening (21.1%), being symptomatic (14.1%), and UTI (5.5%). In 116 children (58.3%), the right kidneys and in 83 (41.7%) the left kidneys were absent. Besides, 14.6% of the participants had consanguineous parents and 3% had a family history of solitary kidney. Upon DMSA scan, the single kidney was scarred in 13.1%, of which only 7.5% were associated with VUR. In addition, proteinuria and hematuria were observed in 6.5% and 1.5% of children, respectively.

**Conclusions:**

The prevalence of reflux nephropathy was 7.5% in children with solitary kidney with a male predominance. Given the relatively high prevalence of reflux nephropathy in these children, screening for VUR in the remnant kidney appears to be essential in this population.

## Introduction

Most solitary kidneys in children are of congenital origin, with renal agenesis accounting for one in every 1000–2000 births [1]. In general, congenital solitary kidney is more common in boys [2, 3].

On the other hand, vesicoureteral reflux (VUR) is the most common urological abnormality in children, mostly diagnosed following a urinary tract infection (UTI) episode. Voiding cystourethrogram (VCUG) is the gold standard for diagnosing VUR, previously routinely performed on every child with a UTI. Nevertheless, VCUG is currently saved for patients at high risk of VUR as recommended by guidelines [4–6].

The renal scarring correlated with VUR is called reflux nephropathy. Reflux nephropathy can be congenital, resulting from the anomalous development of the kidneys causing focal dysplasia in the absence of UTI, or acquired, resulting from renal damage associated with pyelonephritis, with the congenital type more common in males and the acquired type more frequently occurring in females. Dimercaptosuccinic acid scintigraphy (^99m^Tc-DMSA) is considered the gold standard for diagnosing renal scars; however, it cannot differentiate between the scars caused by pyelonephritis in the absence of VUR and congenital or acquired reflux nephropathy [7]. Congenital reflux nephropathy has been reported in 30–60% of children with VUR, mostly diagnosed following the identification of hydronephrosis after birth [8].

No epidemiological studies have been conducted on the prevalence of reflux nephropathy in children with unilateral renal agenesis. Given the importance of the function of the remnant kidney in these children and the significance of timely diagnosis and treatment of reflux nephropathy to prevent further damage, we decided to determine the prevalence of reflux nephropathy in this subgroup of pediatric patients.

## Methods

### Participants

This cross-sectional study included children referred to pediatric nephrologists in different parts of Iran. The inclusion criteria were having DMSA scans and VCUG (standard or isotope). Patients with multicystic dysplastic kidney disease, acquired single kidney due to nephrectomy, and non-functioning remaining kidney were excluded from the study.

### Study design

The study was approved by the institutional review board of Iran University of Medical Sciences (ethics code: IR.IUMS.REC.1399.673) and it complies with the statements of the Declaration of Helsinki. Written informed consent was obtained from the parents/guardians of the participants. A table consisting of the study variables was sent to the members of the Iranian Society of Nephrology. Fourteen centers cooperated and filled out the tables. The reasons for referral included urinary tract infection (UTI), abnormal renal ultrasonography (kidney size, renal outlet obstruction, collecting system dilatation, and parenchymal lesions), being symptomatic, and incidental screening. Demographic characteristics, including age and gender were recorded. History of UTI and presence of vesicoureteral reflux (VUR) were evaluated. Proteinuria was defined as urinary protein excretion at levels higher than 150 mg/m^2^/day. Hematuria was defined as the presence of more than 5 red blood cells (RBCs) per high power field collected in an uncentrifuged mid-stream urine sample. Signs and symptoms of UTI included frequency, dysuria, and urgency in school-aged children, and fever, strong-smelling urine, hematuria, abdominal or flank pain, and new-onset urinary incontinence in infants and younger children. Of note, reflux nephropathy was defined as the presence of renal scarring associated with VUR [9].

### Data analysis

The Statistical Package for the Social Sciences (SPSS) software (version 25.0, Armonk, NY: IBM Corp., USA) was used for data analysis. Mean, standard deviation, median, interquartile range (IQR), frequency, and percentages were used to describe the results. Based on the results of the Kolmogorov–Smirnov test, the independent t-test and the Mann–Whitney test were used to compare quantitative variables between patients with and without reflux nephropathy (among those with VUR). Also, the Fisher’s exact test and the chi-squared test were used to compare qualitative variables between these two groups of patients. *P*-values < 0.05 were regarded as statistically significant.

## Results

Of the 274 children included in this study with the mean age of 4.71 ± 4.24 years, 199 (72.6%) had unilateral renal agenesis. General characteristics of the study population based on availability of data are presented in Table 1.

**Table 1:** General characteristics of the study population

Variable	Number of patients included	Minimum	Maximum	Mean ± SD
Age (years)	274	0	17	4.71 ± 4.24
Birth weight (kg)	142	0.9	7.25	3.21 ± 0.69
Birth height (cm)	90	38	54	48.85 ± 2.34
Basal Cr (mg/dl)	183	0.3	2.9	0.61 ± 0.27
Current height (cm)	213	2	80	22.77 ± 15.34
Current weight (kg)	126	44	175	106.43 ± 31.08
eGFR (ml/min/1.73 m^2^)	90	35.5	176	93.49 ± 26.79

*Abbreviations*: *SD* standard deviation, *Cr* creatinine, *eGFR* estimated glomerular filtration rate

Among children with unilateral renal agenesis, 118 (59.3%) were male and 81 (40.7%) were female. In 116 children (58.3%), the right kidney and in 83 (41.7%) the left kidney was absent. In general, the most common cause of referral was abnormal renal ultrasonography (40.2%), followed by incidental screening (21.1%), being symptomatic (14.1%), and UTI (5.5%). Besides, 21.1% of these children had a history of UTI and 23.1% were diagnosed with VUR (Table 2). Moreover, 14.6% of the participants had consanguineous parents and 3% had a family history of solitary kidney. Upon DMSA scan, scarring of the single kidney was observed in 26/199 patients (13.1%), of which only 15 (7.5%) were associated with VUR and correspond to the definition of reflux nephropathy. In addition, proteinuria and hematuria were observed in 6.5% and 1.5% of children, respectively.

**Table 2:** Comparison of gender, cause of referral, history of UTI, and VUR between children with right-sided and left-sided absent kidney

Variables	Absent kidney N (%)		Total N (%)
	Right (*N* = 116)	Left (*N* = 83)	
**Gender**			
Male	70 (60.3)	48 (57.8)	118 (59.3)
Female	46 (39.7)	35 (42.2)	81 (60.7)
**Cause of referral**			
UTI	4 (3.4)	7 (8.4)	11 (5.5)
Abnormal RUS	52 (44.8)	28 (33.7)	80 (40.2)
Symptomatic	16 (13.8)	12 (14.5)	28 (14.1)
Incidental screening	27 (23.3)	19 (22.9)	46 (23.1)
Unknown	17 (14.7)	17 (20.5)	34 (17.1)
**History of UTI**			
Yes	26 (22.4)	16 (19.3)	42 (21.1)
No	88 (75.9)	64 (77.1)	152 (76.4)
Unknown	2 (1.7)	3 (3.6)	5 (2.5)
**VUR**			
Yes	32 (27.6)	14 (16.9)	46 (23.1)
No	60 (51.7)	43 (51.8)	103 (51.8)
**Consanguineous parents**			
Yes	16 (13.8)	13 (16.7)	29 (14.6)
No	64 (55.2)	43 (51.8)	107 (53.8)
Unknown	36 (31)	27 (31.5)	63 (31.6)
**Family history of solitary kidney**			
Yes	2 (1.7)	4 (4.8)	6 (3)
No	10 (8.6)	16 (19.3)	26 (13.1)
Unknown	104 (89.7)	63 (75.9)	167 (83.9)
**DMSA scan**			
Scarred kidney	16 (13.8)	10 (12)	26 (13.1)
Normal kidney	99 (85.3)	71 (85.5)	170 (85.4)
**Proteinuria**			
Yes	6 (5.2)	7 (8.4)	13 (6.5)
No	58 (50)	42 (50.6)	100 (50.3)
**Hematuria**			
Yes	2 (1.7)	1 (1.2)	3 (1.5)
No	77 (66.4)	56 (67.5)	133 (66.8)
**Outcome**			
CKD			17
rUTI			6
Normal kidney function			164
Lost to follow-up			20

*Abbreviations*: *N* number, *UTI* urinary tract infection, *VUR* vesicoureteral reflux, *RUS* renal ultrasonography, *DMSA* dimercaptosuccinic acid, *CKD* chronic kidney disease, *rUTI* recurrent UTI

In children with VUR (*n* = 46), the presence of reflux nephropathy was not correlated with age, gender, birth weight and height, current weight and height, eGFR, history of UTI, VUR grade, having consanguineous parents, or family history of solitary kidney (Table 3).

**Table 3:** Correlation of reflux nephropathy with different factors in patients with VUR (*n* = 46)

Variable	With RN	Without RN	*P*-value*
Age (years) median (IQR)	4.50 (1.40 – 7.80)	3.00 (0.32 – 6.10)	0.301
Gender N (%)			
Male	12 (42.9)	16 (57.1)	0.064^a^
Female	3 (16.7)	15 (83.3)	
Birth weight (kg) median (IQR)	3.02 (2.75 – 3.18)	3.08 (2.89 – 3.25)	0.458
Birth height (cm) mean ± SD	48.36 ± 2.01	49.19 ± 1.60	0.248^b^
Basal Cr (mg/dl) median (IQR)	0.53 (0.45 – 0.65)	0.57 (0.50 – 0.68)	0.363
Current height (cm) median (IQR)	100.00 (82.50 – 113.50)	98.00 (86.25 – 119.25)	0.927
Current weight (kg) median (IQR)	17.33 (13.40 – 24.25)	15.90 (10.92 – 21.90)	0.429
eGFR (ml/min/1.73 m^2^) mean ± SD	74.11 ± 12.50	96.23 ± 33.98	0.182^b^
History of UTI N (%)			
Yes	7 (31.8)	15 (68.2)	0.466^c^
No	7 (30.4)	16 (69.6)	
Unknown	1 (100.0)	0 (0.0)	
VUR grade N (%)			
I-III	9 (34.6)	17 (65.4)	0.434^c^
IV-V	5 (41.7)	7 (58.3)	
Unknown	1 (12.5)	7 (87.5)	
Consanguineous parents N (%)			
Yes	0 (0.0)	9 (100.0)	0.063^a^
No	9 (42.9)	12 (57.1)	
Unknown	6 (37.5)	10 (62.5)	
Family history of solitary kidney N (%)			
Yes	0 (0.0)	1 (100.0)	0.202^c^
No	0 (0.0)	5 (100.0)	
Unknown	15 (37.5)	25 (62.5)	

*Abbreviations*: *N* number, *SD* standard deviation, *IQR* interquartile range, *RN* reflux nephropathy, *Cr* creatinine, *eGFR* estimated glomerular filtration rate, *UTI* urinary tract infection, *VUR* vesicoureteral reflux

^*^Analyzed by the Mann–Whitney test

^a^Analyzed by the chi-squared test

^b^Analyzed by the independent t-test

^c^Analyzed by the Fisher’s exact test

## Discussion

The reduction in renal mass due to unilateral renal agenesis can lead to compensatory hypertrophy of the contralateral kidney [10, 11]. With regard to the function of the remnant kidney, it is crucial to determine whether this hypertrophy is associated with an increased number of nephrons or enlargement of the existing nephrons. It has been demonstrated in animal studies that compensatory nephrogenesis in the contralateral kidney, leading to an increase in the number of nephrons, is responsible for its enlargement [10, 12]. However, no human studies have reported similar results, yet better long-term glomerular filtration rates have been observed in children with congenital solitary kidney compared to those with acquired solitary kidney [11].

To the best of our knowledge, the prevalence of reflux nephropathy in children with solitary kidney has not been investigated in previous studies and this is the first report in this regard. VUR was present in 23.1% of children with solitary kidney in this study. The significance of VUR relies on its association with renal scarring and chronic kidney disease (CKD) because it predisposes children to recurrent UTIs and the subsequent ascending infection, leading to destruction and scarring of the renal parenchyma [13]. VCUG is the “gold standard” for the detection of VUR and not only does it detect VUR but also it allows grading of its severity. However, due to exposure to radiation, invasiveness of the procedure, and the expenses, VCUG is only indicated when medical reasons necessitate the performance of the test [14]. This is even more crucial when it comes to children with solitary kidney; however, it does not negate the need for such evaluation in these patients. On the other hand, congenital renal anomalies, rather than UTIs, are the primary causes of renal injury in solitary kidneys, since VUR is associated with renal damage to solitary kidneys due to the genetic-related dysplasia regardless of UTIs [15–18]. Nevertheless, Marzuillo and Polito have argued against the performance of VCUG in all children with solitary kidney, as the detection of VUR by VCUG only presented useful prognostic information in 0.9% of their patients [19].

The cause of referral for further evaluations was abnormal RUS findings in 40.2% of the participants in the current study. Yamamoto et al. showed that the only risk factor for abnormal VCUG was abnormal RUS findings [20]; nonetheless, their study included children with unilateral multicystic dysplastic kidney. The majority of congenital kidney and urinary tract anomalies can be detected by RUS; however, 2/3 cases of VUR are not detected by RUS even when significant VUR is present [21], which confirms the need for VCUG in the detection of VUR. Therefore, abnormal RUS findings cannot be the only reason for further investigation in children with solitary kidney.

CKD is currently a significant health issue globally. It is considered a risk factor for all-cause mortality as well as progression to end-stage renal disease [22]. In a large cohort of Korean adults, solitary kidney was an independent risk factor for CKD, yet this association was stronger in patients with acquired solitary kidney compared to those with congenital solitary kidney [23]. In our study, proteinuria and hematuria were observed in 6.5% and 1.5% of children with unilateral renal agenesis, respectively which can be indicators of kidney damage leading to CKD. In order to determine the true burden of VUR in children with solitary kidney, the patients of the current study are required to be followed for long periods of time. Moreover, in the study by La Scola et al. children with congenital solitary kidney were at increased risk of hypertension compared to those with two kidneys [24] implicating the necessity of screening for hypertension in this population.

The major strength of the current study was the evaluation of patients using DMSA scans, which enabled us to differentiate between VUR and reflux nephropathy. Another strength was the multi-centric design of the study. Also, to the best of our knowledge, this is the first study evaluating the prevalence of reflux nephropathy in children with solitary kidney. Nonetheless, our study was not without limitations. One limitation was that due to the multi-centric design of the study, imaging and laboratory evaluations had been made at different centers, with potential differences in the accuracy of measurements and interpretations. Another limitation was our relatively small sample size, which prompts cautious generalization of the findings.

## Conclusions

The prevalence of reflux nephropathy was 7.5% in children with solitary kidney with a male predominance. Given the relatively high prevalence of reflux nephropathy in these children, screening for VUR in the remnant kidney appears to be essential in this population. Early diagnosis of VUR in children with unilateral kidney agenesis can prevent the development of permanent damage to the remaining kidney.

## Data Availability

The datasets used and/or analyzed during the current study are available from the corresponding author on reasonable request.

## Abbreviations

DMSA: Dimercaptosuccinic acid
UTI: Urinary tract infection
VCUG: Voiding cystourethrogram
VUR: Vesicoureteral reflux
CKD: Chronic kidney disease
